# Development of *Phytomonas lipae* sp. n. (Kinetoplastea: Trypanosomatidae) in the true bug *Coreus marginatus* (Heteroptera: Coreidae) and insights into the evolution of life cycles in the genus *Phytomonas*

**DOI:** 10.1371/journal.pone.0214484

**Published:** 2019-04-03

**Authors:** Alexander O. Frolov, Marina N. Malysheva, Anna I. Ganyukova, Viktoria V. Spodareva, Vyacheslav Yurchenko, Alexei Y. Kostygov

**Affiliations:** 1 Zoological Institute of the Russian Academy of Sciences, St. Petersburg, Russia; 2 Life Science Research Centre, Faculty of Science, University of Ostrava, Ostrava, Czech Republic; 3 Martsinovsky Institute of Medical Parasitology, Tropical and Vector Borne Diseases, Sechenov University, Moscow, Russia; 4 Institute of Environmental Technologies, Faculty of Science, University of Ostrava, Ostrava, Czech Republic; Instituto Oswaldo Cruz, BRAZIL

## Abstract

Here we described a new trypanosomatid species, *Phytomonas lipae*, parasitizing the dock bug *Coreus marginatus* based on axenic culture and *in vivo* material. Using light and electron microscopy we characterized the development of this flagellate in the intestine, hemolymph and salivary glands of its insect host. The intestinal promastigotes of *Phytomonas lipae* do not divide and occur only in the anterior part of the midgut. From there they pass into hemolymph, increasing in size, and then to salivary glands, where they actively proliferate without attachment to the host's epithelium and form infective endomastigotes. We conducted molecular phylogenetic analyses based on 18s rRNA, gGAPDH and HSP83 gene sequences, of which the third marker performed the best in terms of resolving phylogenetic relationships within the genus *Phytomonas*. Our inference demonstrated rather early origin of the lineage comprising the new species, right after that of *P*. *oxycareni*, which represents the earliest known branch within the *Phytomonas* clade. This allowed us to compare the development of *P*. *lipae* and three other *Phytomonas* spp. in their insect hosts and reconstruct the vectorial part of the life cycle of their common ancestor.

## Introduction

The family Trypanosomatidae is a group of obligate parasitic flagellates, whose evolution was mainly shaped by the exploration of various animals [1]. Monoxenous (with one host in the life cycle) representatives are known as worldwide dispersed parasites of insects [2,3]. The majority of dixenous (with two hosts in their life cycle) trypanosomatids live in vertebrates and use insects and, less frequently, leeches as vectors [4]. This concerns genera *Trypanosoma* and *Leishmania*, among which there are agents of numerous important diseases of human (e.g. sleeping sickness, Chagas diseases, kala-azar, espundia, oriental sore, etc.), as well as wild and domestic animals (e.g. nagana, surra, or dourine) [5–7].

The members of *Phytomonas*, another dixenous genus, represent a notable exception in the evolutionary trend of trypanosomatids. These peculiar flagellates adapted to parasitism in various plants, thereby significantly expanding the host range of the family [8,9]. The first species of this genus was described as *Leptomonas davidi* more than a century ago from the latex of the spurge *Euphorbia hirta* in Mauritius by Alexandre Lafont [10]. Later in the same year, Charles Donovan found this species in the same plant in Madras and established the new genus *Phytomonas* for it [11]. Soon after, Lafont demonstrated that parasite is transmitted by the phytophagous heteropteran *Nysius euphorbiae* (Lygaeidae) [12]. *Phytomonas* spp. were later discovered in the phloem, fruits, and flowers of members of more than 20 different families of the vascular plants, as well as in phytophagous true bugs of 3 families (Coreidae, Pentatomidae, and Lygaeidae) [8,9,13–16].

The vast majority of the members of this genus are represented by over 200 isolates, for which the available information is mostly restricted to either plant or insect species they were isolated from, and, occasionally, GenBank sequences [15]. While about 15 nominal species of this genus were described in the pre-molecular era [3,8], only 6 of those (*Phytomonas serpens*, *P*. *françai*, *P*. *mcghee*, *P*. *nordicus*, *P*. *oxycareni*, and *P*. *dolleti*) have been assessed using molecular phylogenetic methods [17–21]. Whole genomes of *P*. *françai*, *P*. *serpens* and two undescribed *Phytomonas* sp. isolates (EM1 and HART1) have been recently published [22–24].

The monophyly of the genus *Phytomonas* has been confirmed in several molecular phylogenetic and phylogenomic studies [4,19,21,23,25]. However, in many respects, this group of flagellates is quite heterogeneous. The intrageneric clades, revealed using various molecular markers, usually do not correlate with either host tropism, ecology or biogeography [17,20,26,27]. This phenomenon has not been explained so far, but an unbiased analysis is not possible because of uneven exploration of phytomonads from different host groups and geographic regions [15]. Only about one quarter of all described isolates came from Europe, Asia, Africa, or Australia/Oceania, while the majority has American origin [15]. Because of the economic importance, the research has always been biased towards parasites infecting agricultural plants (coconut and oil palms, edible fruits, coffee and cacao trees, cassava, etc.). At the same time, the knowledge of *Phytomonas* spp. real diversity and biogeography remained fragmented. One of such uncharted territories is northern Eurasia. The only species described north of the 50° N is secondarily monoxenous *P*. *nordicus* from the predatory true bug *Troilus luridus*. Its life cycle is aberrant and does not involve plant host [19,28].

In 1966, Jerzy Lipa described *Blastocrithidia raabei* from the dock bug *Coreus marginatus* (Heteroptera: Coreidae) in Białowieża National Park, Poland. He reported presence of long (up to 41 μm) leptomonads (= promastigotes) in the anterior part of the intestine and, less frequently, in the hemolymph of the bugs infected by this trypanosomatid. Lipa considered these cells as developmental stages of *B*. *raabei* [29]. However, the promastigote stage is not intrinsic to *Blastocrithidia* spp. and their life cycles are not known to include development in hemolymph [30–32]. Thus, the most likely explanation is that Lipa observed mixed infection of *B*. *raabei* with a trypanosomatid of another genus.

Here, using light and electron microscopy, we described a new trypanosomatid inhabiting intestine, hemolymph and salivary glands of the dock bugs from European and Asian localities in Russia. Similarly to the unidentified flagellate from Lipa's description, the main developmental stage of this species is a promastigote. Our molecular phylogenetic analyses proved that the new species belongs to the genus *Phytomonas* and we named it after Jerzy Lipa–*Phytomonas lipae*. To date, this is the fourth member of this genus with characterized development in the insect vector.

## Material and methods

### Hosts

The dock bugs *Coreus marginatus* were collected in 2016–2018 from vegetative and generative parts of the Russian dock *Rumex confertus*, the bitter dock *R*. *obtusifolius*, and the false rhubarb *Rheum rhaponticum* (the two latter plant species were not present in the Asian location, see below). The bugs originated from the North-West of the European part of Russia (Pskov Oblast, village Lyady, 58°35' N, 28°55' E; and Novgorod Oblast, village Oksochi, 58°39' N, 32°47' E) and one Asian population in the south of Western Siberia (Kurgan Oblast, village Zaozerny, 55°28' N, 65°16' E). The bugs were studied from May to September: 78 imagines and 36 nymphs in total from the first and second localities and 26 imagines from the third one.

**Fig 1:**
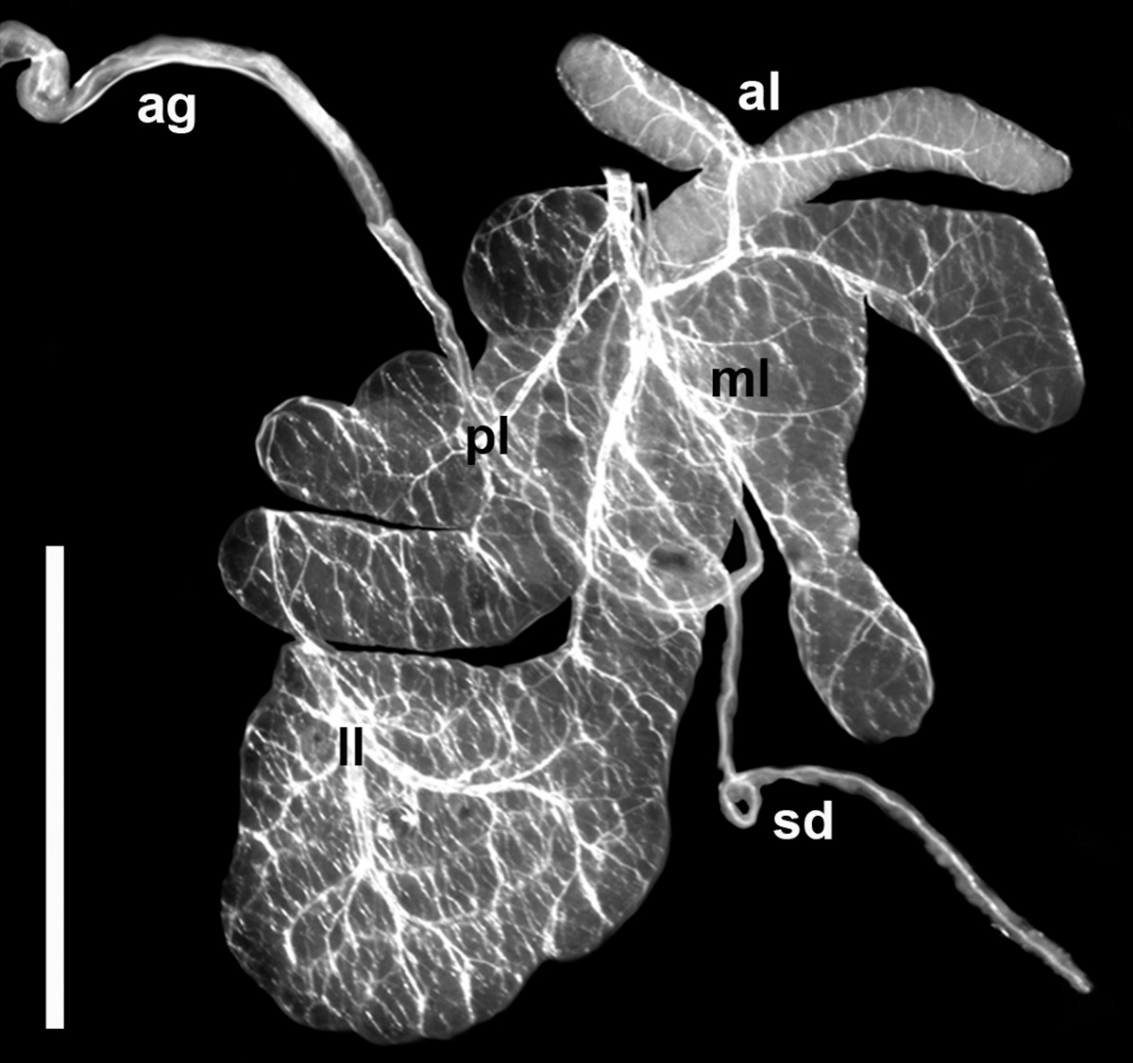
Isolated salivary gland of *Coreus marginatus* (*ex vivo*, reflected light). **ag**–accessory Salivary gland; **al**–anterior lobe of the principal Salivary gland; **ll–**lateral lobe of the principal Salivary gland; **ml–**median lobe of the principal Salivary gland; **pl**–posterior lobe of the principal Salivary gland; **sd**–principal duct of the Salivary gland. Scale bar 3 mm.

The bugs were dissected in normal saline solution (Fig 1) under LOMO MBS-2 stereomicroscope (Micromed, Russia) as described previously [33]. Hemolymph and salivary gland samples were prepared and processed as in [19].

The adult lime seed bugs *Oxycarenus lavaterae*, hosts of *P*. *oxycareni*, were collected in October 2018 when they formed large aggregates on the linden tree trunks in the Komenského Sady park (49°51' N, 18°17' E) in Ostrava, Czech Republic. They were not dissected, but pooled, smashed with pipette tips and used directly for DNA isolation.

No specific permissions were required for the insects' sampling, since the localities, where they were collected, are of public access and neither *Coreus marginatus* nor *Oxycarenus lavaterae* are endangered or protected species.

### Cultivation and cryopreservation of trypanosomatids

Sixteen primary (xenic) cultures from the gut and salivary glands of the infected *C*. *marginatus* individuals were established in several media: Brain Heart Infusion, Schneider's *Drosophila* Medium, TC-100 Insect Medium, RPMI 1640, and M199 (all from Sigma-Aldrich, St. Louis, MO, USA) as well as overlaid blood agar all supplemented with 10% of the fetal bovine serum (FBS) (BioloT, St. Petersburg, Russia), 500 μg/ml of streptomycin and 500 Units/ml of penicillin (Sigma-Aldrich). Purification of the cultures from fungal contaminants was conducted using a device described before [34]. Axenic cultures were kept at 20º C and passaged monthly. Their cells were cryopreserved in the growth media supplemented with 10% DMSO (Sigma-Aldrich) and stored at -86º C.

### Microscopy

The smears from infected intestine and salivary glands were fixed for 30 min with ethanol and stained with either Giemsa or 4’,6-diamidino-2-phenylindole (DAPI) as described before [35,36]. Digital images were acquired in DM 2500 microscope (Leica Microsystems GmbH, Wetzlar, Germany) equipped with UCMOS14000KPA 14-Mpx camera (Toup Tek, Hangzhou, China) at ×1,000 magnification. All measurements of cells (n = 25) and statistical analysis were performed in UTHSCSA Image Tool for Windows v. 3.0. For transmission and scanning electron microscopy the samples were fixed and processed as described previously [37].

### DNA isolation, amplification, cloning, and sequencing

Whole bodies of thirty lime seed bugs (sample Ox1), infected salivary glands (samples Cor8sg, Cor 40sg, Cor203sg) and intestine (sample Cor8i) of dock bugs, as well as cultured cells (samples Cor 4, Cor 49, Cor203) were used for total genomic DNA isolation with the DNeasy Blood & Tissue Kit (Qiagen, Hilden, Germany) according to the manufacturer’s instructions. In addition to these, the laboratory cell cultures of *Lafontella* sp. GMO-01 [38], *Herpetomonas samuelpessoai* ATCC 30252, and *H*. *muscarum* ATCC 30260 were used for DNA isolation and amplification of particular molecular markers.

The ITS1-5.8S-ITS2 region and nearly full-length small SSU rRNA, gGAPDH, and HSP83 genes were amplified using the respective primer pairs: IAMWE and IRBAB [27], S762 and S763 [39], M200 and M201 [40], as well as 100XF and 970XR [41]. With the exception of the ITS1-5.8S-ITS2 fragment, which was first cloned using the InsTA PCR Cloning Kit (Thermofisher Scientific, Waltham, USA), all other amplicons were sequenced directly with the amplification primers. In addition, the internally annealing oligonucleotides 883F, 907R S757, and A757 [42], as well as XF2 and XR2 [43] were used for the sequencing of SSU rRNA and HSP83 genes respectively. The GenBank accession numbers for the new sequences determined in this work are: MK036047 –MK036051, MK249803 (SSU rRNA gene of *P*. *lipae* isolates Cor4, Cor8sg, Cor8i, Cor40sg, Cor49, and *P*. *oxycareni*, respectively); MK050458 –MK050461, MK258194, MK258195 (gGAPDH gene of *P*. *lipae* isolates Cor4, Cor8sg, Cor40sg, and Cor49, *P*. *oxycareni*, and *Lafontella* sp. GMO-01, respectively); MK053634 (ITS1-5.8S-ITS2 fragment of Cor8sg); and MK258188 –MK258193 (HSP83 gene of *Herpetomonas nabiculae*, *H*. *muscarum*, *H*. *samuelpessoai*, *P*. *lipae* isolate 49Cor, *Lafontella* sp. GMO-01, and *P*. *oxycareni*, respectively).

### Phylogenetic analyses

The sequences of SSU rRNA, gGAPDH and HSP83 genes obtained in this study were combined with those available in the GenBank (nr and wgs databases [44]). The alignment of SSU rRNA gene sequences was performed in MAFFT 7.4 with the E-INS-i algorithm [45]. The datasets for gGAPDH and HSP83 genes were processed in MEGA7 [46] as follows: translated into amino acids, aligned with the built-in Muscle module [47] and then reverse translated to nucleotides. Taking into account that the SSU rRNA and HSP83 alignments contained ambiguously aligned positions, they were trimmed using Gblocks v. 0.91 [48], as previously described [49]. However, the presence of many short sequences in the SSU rRNA dataset resulted in an excessive end trimming and, consequently, removal of significant amount of phylogenetic information. In order to avoid this, the trimming mask was obtained on the subset of sequences longer than 1,800 bp and then applied to the whole dataset. The final lengths of the three resulting alignments were 2,106; 1,089; and 1,909 bp for SSU rRNA, gGAPDH, and HSP83 genes, respectively. The ITS1-5.8S-ITS2 fragment was not used for the phylogenetic analyses, since this marker provides resolution only in terminal branches, whereas determining the phylogenetic position of *P*. *lipae* required deep-level resolution (see Results).

The maximum likelihood and Bayesian tree reconstructions were performed in IQ-TREE v.1.68 [50] and MrBayes v.3.2.6 [51], respectively, with partitioning of protein-coding genes by codon position as described before [52]. The GenBank accession numbers of the sequences used in all analyses are listed in S1 Table.

## Results

### Prevalence of trypanosomatids' infection in *Coreus marginatus*

Of 78 imagines and 36 nymphs of the dock bug *C*. *marginatus*, collected in the Northwest Russia, 7 (~ 9%) and 2 (~ 6%) specimens contained promastigotes in their salivary glands. Of 11 imagines from Novgorod Oblast, that were dissected within the first week after appearance on the plants, 2 bugs (~ 18%) had trypanosomatid infection in salivary glands. A significant proportion of the analyzed *C*. *marginatus* imagines (~ 40%, 31 out of 78) were infected with *Blastocrithidia raabei* (identified by characteristic features and morphometry of its epimastigotes [29]). Of these, mixed infections by *B*. *raabei* and the species under study were documented in four cases (~ 13%). The analysis of the Asian population (Kurgan Oblast) revealed promastigotes in salivary glands of a single imago out of 26 dissected (~ 4%).

### Cultivation of trypanosomatids

The flagellates from the gut and salivary glands of the infected *C*. *marginatus* were cultivated in several different media. In all cases, the promastigotes could be maintained in xenic (fungi-contaminated) cultures. The cells were also viable in purified axenic cultures, but usually they did not divide. Out of sixteen original cultures, only three (all from salivary glands) were successfully established in axenic conditions. In two of these (Cor4 and Cor49, from Novgorod Oblast), the promastigotes started division in the FBS-supplemented TC-100 Insect Medium almost 1 year after purification. The culture Cor203 (from Kurgan Oblast) was obtained in the same way, but the cells started division after the second passage.

### Phylogenetic analyses

**Fig 2:**
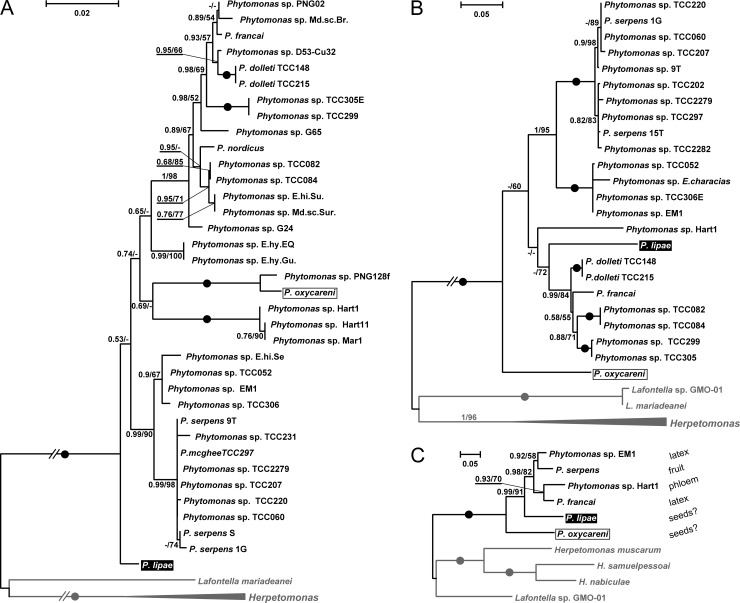
Maximum likelihood phylogenetic trees reconstructed using different molecular markers. **A**, SSU rRNA; **B**, gGAPDH**; C**, HSP83. Numbers at nodes indicate posterior probability and bootstrap percentage, respectively. Values less than 0.5 and 50% are replaced with dashes. Nodes having 1.0 posterior probability, 100% bootstrap support are marked with black circles. Double-crossed branches are at 50% of their original lengths. The trees are rooted with the sequences of *Herpetomonas* and *Lafontella spp*. (shown in grey). The scale bar represents number of substitutions per site. *Phytomonas lipae* is highlighted and *P*. *oxycareni* is boxed. The known/supposed parasitized plant host organs and tissues are indicated in **C**.

The phylogenetic inferences based on SSU rRNA and gGAPDH genes, the two molecular markers traditionally used for trypanosomatids [53], demonstrated low resolution in the basal part of the *Phytomonas* clade (Fig 2). Thus, it was not possible to reliably determine the phylogenetic position of *P*. *lipae*. On both maximum likelihood and Bayesian trees of the SSU rRNA gene, the new species appeared as the earliest branch within the genus (Fig 2A). However, this topology was poorly supported. Moreover, *P*. *oxycareni*, which has been previously shown to occupy this position [21], was placed elsewhere (also with low statistical support). Removal of various taxa from the dataset demonstrated that the position of both species on the tree was unstable. A similar situation was observed for the gGAPDH gene (Fig 2B), but in this case, there was a discrepancy between maximum likelihood and Bayesian trees, which placed (with low statistical support) as the earliest branch either *P*. *oxycarenus* or *P*. *lipae*, respectively.

Taking into account these difficulties, we switched to HSP83 gene, which has recently proved to be efficient in resolving phylogenetic relationships in a different trypanosomatid group [43]. We created a dataset containing the representatives of all key lineages of *Phytomonas* spp. and inferred a phylogenetic tree with substantially better supports. Both maximum likelihood and Bayesian analyses based on this gene demonstrated well-supported positions of *P*. *oxycarenus* and *P*. *lipae* as the first and the second early branches within the clade of *Phytomonas*. Importantly, in relation to the former species, this agrees with the earlier SSU rRNA gene-based inferences [21].

### Morphology of flagellates

Promastigotes of *P*. *lipae* were found mainly within the principal salivary glands of *C*. *marginatus* (Fig 1), where they infected all four lobes. No parasites were detected in accessory salivary glands or principal duct of the Salivary gland. In three infected bugs, phytomonad cells were also found in M1 and M2 segments of the intestine, and in one specimen, a few cells were observed in hemolymph.

**Fig 3:**
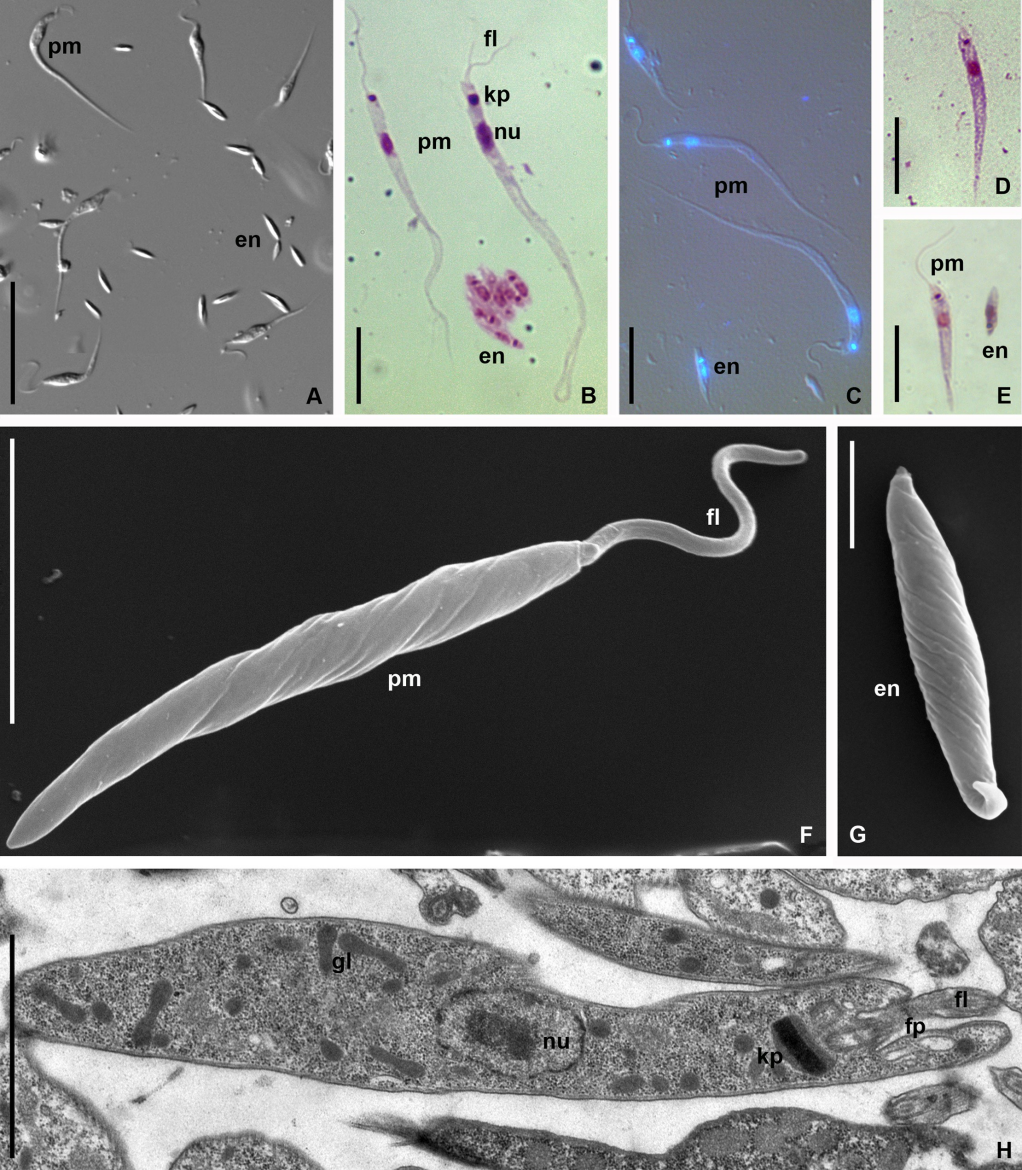
Morphology of *Phytomonas lipae* (light microscopy, SEM and TEM). **A—C**. *Phytomonas lipae* in the salivary gland of *C*. *marginatus*; **D.** Promastigote of *P*. *lipae* from the M1 segment of the host's midgut; **E—H**. *P*. *lipae* in the axenic culture Cor4. **A–***ex vivo*, DIC; **B, D, E–**Giemsa; **C**–overlaid DIC and DAPI; **F**, **G**–SEM, **H**–TEM. **en**–endomastigote; **fl**–flagellum; **fp–**flagellar pocket**; gl**–glycosomes; **kp**–kinetoplast; **nu–**nucleus; **pm–**promastigote. Scale bars 20 μm (**A**); 10 μm (**B—E**); 5 μm (**F**); 2 μm (**G, H**).

The parasites in the salivary glands of the bugs from European and Asiatic populations were similar (Table 1, S2 Table). There were two main morphotypes (Fig 3A–3C): i) large (up to 70 μm, but mainly 30–40 μm long) slim promastigotes with short flagellum and elongated whip-like posterior end (Table 1, S2 Table); ii) small (~ 8 μm long) endomastigotes (Fig 3A–3C, Table 1, S2 Table). In addition to these two morphotypes, there was a continuum of intermediate cells in the micropopulations of parasites infecting salivary glands. The large promastigotes were usually twisted with one or several turns. Both nucleus and kinetoplast were localized to the anterior third of the cell at distance comparable to the nucleus length (Fig 3B and 3C). The flagellum was short, about 1/3–1/4 of the cell body (Table 1, S2 Table).

**Table 1:** Morphometry of different cell types of *P*. *lipae* from culture, different populations and different organs of the hosts (N = 25).

	Length	Width	Flagellum	Nucleus	N-A	K-A	N-K
**Promastigotes in the salivary glands**							
Cor4sg (hapantotype), Novgorod Oblast	36.4 ± 10.5 (13.7–66.3)	1.9 ± 0.4 (1.4–3.2)	9.0 ± 1.7 (5.9–12.0)	2.5 ± 0.5 (1.7–3.5)	6.6 ± 1.6 (4.3–9.0)	1.7 ± 0.6 (1.5–2.7)	2.9 ± 1.1 (0.2–4.6)
Cor203sg, Kurgan Oblast	34.2 ± 10.2 (12.5–56.0)	1.6 ± 0.3 (1.1–2.3)	7.2 ± 2.1 (4.4–11.7)	1.9 ± 0.3 (1.4–2.8)	4.2 ± 1.0 (3.0–6.2)	1.4 ± 0.5 (0.8–2.3)	1.8 ± 1.1 (0–2.7)
**Endomastigotes in the salivary glands**							
Cor4sg (hapantotype), Novgorod Oblast	8.2 ± 1.8 (6.8–12.0)	1.5 ± 0.2 (1.1–1.7)	N/A	1.7 ± 0.2 (1.4–3.2)	2.9 ± 0.2 (2.4–4.0)	0.9 ± 0.2 (0.1–1.3)	0.04 ± 0.11 (0–0.26)
Cor203sg, Kurgan Oblast	7.7 ± 1.4 (6.4–11.3)	1.4 ± 0.2 (1.1–1.6)	N/A	1.7 ± 0.3 (1.3–2.3)	2.7 ± 0.3 (2.2–3.2)	0.8 ± 0.2 (0.3–1.2)	0.03 ± 0.06 (0–0.19)
**Promastigotes in the M1 intestinal segment**							
Cor4_M1, Novgorod Oblast	20.9 ± 4.8 (16.1–28.3)	1.5 ± 0.2 (1.2–1.9)	20.3 ± 3.1 (12.7–23.4)	2.5 ± 0.7 (1.0–3.2)	6.6 ± 1.9 (4.1–9.8)	1.8 ± 0.5 (0.8–2.4)	3.4 ± 1.4 (1.4–5.5)
**Promastigotes in the culture**							
Cor4, culture	17.0 ± 2.7 (13.9–21.2)	2.1 ± 0.3 (1.6–2.6)	8.7 ± 2.6 (3.5–12.6)	2.1 ± 0.2 (1.6–2.5)	5.4 ± 0.9 (4.2–7.8)	1.4 ± 0.4 (0.3–1.8)	2.0 ± 0.9 (0.9–4.5)

N-A is the distance between the nucleus and the anterior end of the cell. K-A is the distance between the kinetoplast and the anterior end of the cell. N-K is a distance between the nucleus and the kinetoplast. All the measurements are in μm.

Promastigotes found in the midgut were smaller, usually under 30 μm long and had not whip-like posterior ends (Fig 3D, Table 1, S2 Table). The localization of nucleus and kinetoplast was similar to that of the salivary gland forms. Midgut promastigotes had longer flagella, comparable to the cell body in length. (Table 1).

In all three cultures isolated from bugs' salivary glands (Cor4, Cor49, Cor203), flagellates were morphologically similar and included promastigotes and small aflagellated cells (Fig 3E3–3G). Promastigotes in the cultures resembled those from the midgut, but were slightly shorter and their flagella length was roughly ½ of that of the cell body (Table 1, S2 Table). Large promastigotes, similar to those from the salivary glands, were rare (~0.01%) and appeared only after a prolonged cultivation.

Ultrastructural organization of all studied promastigote types was similar both in salivary glands and in cultures. Kinetoplast was compact (diameter 0.67 ± 0.13 μm, thickness 0.18 ± 0.06 μm). As in many other trypanosomatids, the Golgi apparatus was located between kinetoplast and nucleus. The cytoplasm displayed multiple glycosomes profiles. The flagellar pocket was short and opened terminally (Fig 3H).

### Ultrastructure of host-parasite relationships in the salivary glands

**Fig 4:**
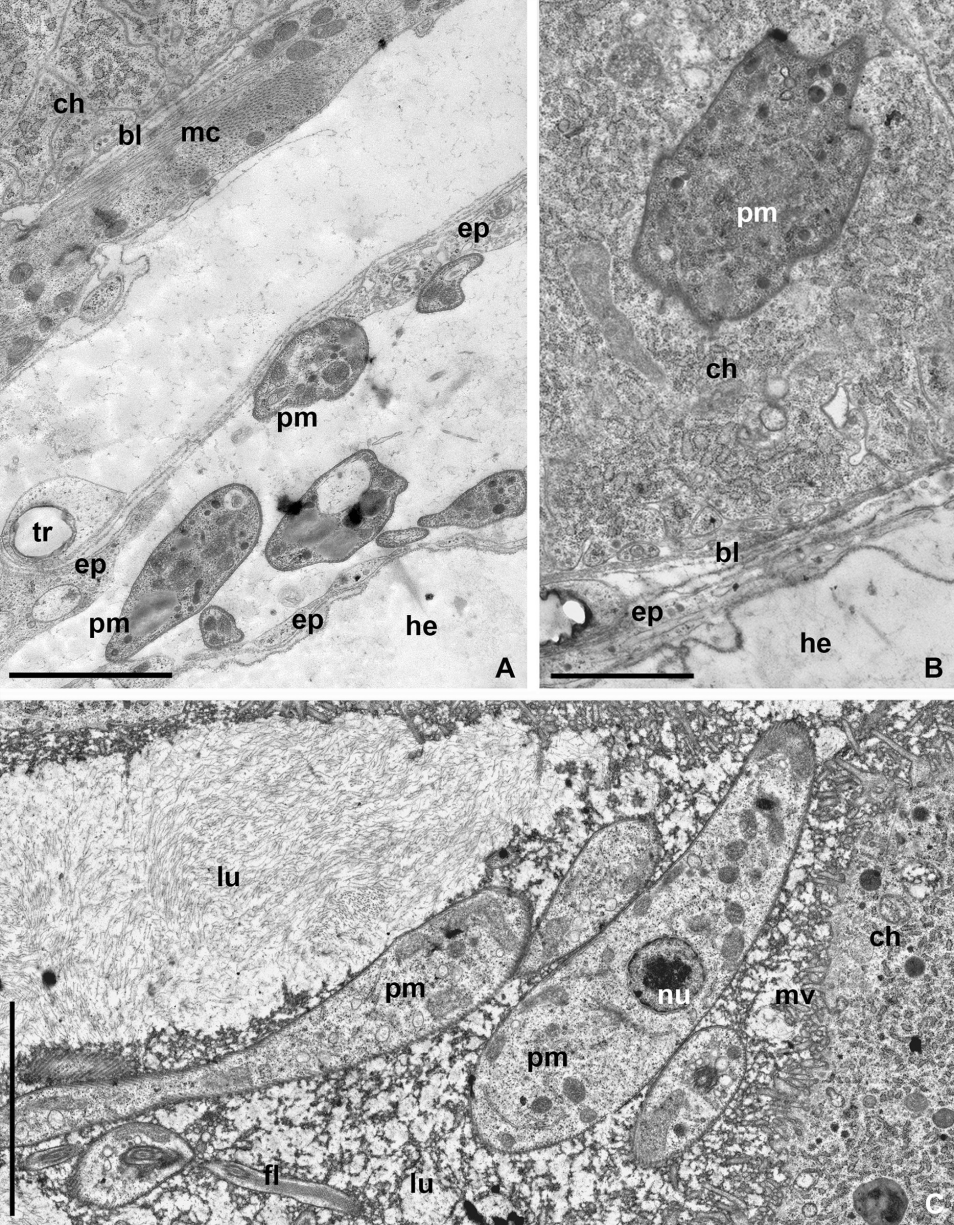
*Phytomonas lipae* in the salivary glands of *C*. *marginatus* (TEM). **A.–**Promastigotes of *P*. *lipae* in the coelomic epithelium; **B.** *P*. *lipae* in the cytoplasm of a salivary gland's cell; **C–**Promastigotes in the lumen. **bl**–basal lamina; **ch**–host's salivary gland cell; **ep**–coelomic epithelium; **he**–hemocoel; **lu**–lumen of the salivary gland; **mc**–muscle cells; **mv**–microvilli; **tr**–trachea. Other abbreviations are as in Fig 3. Scale bars: 2 μm (**A, C**); 2.5 μm (**B**).

The parasites were detected in the squamous coelomic epithelium, separating the salivary glands from the hemocoel, as well as in the cuboidal epithelium and lumen. In the coelomic epithelium, promastigotes were situated in lacunae between epitheliocytes, tracheae and myocytes (Fig 4A). Transient stages could be observed in the cytoplasm of the cuboidal epithelial cells (Fig 4B) as single promastigotes enclosed in parasitophorous vacuoles. The vast majority of promastigotes localized to the gland lumen in the space between secretory granules. There they actively divided, forming aggregates on the gland surface, but not attaching to the microvilli of the host cell by their flagella (Fig 4C).

### Taxonomic summary

**Class:** Kinetoplastea (Honigberg, 1963) Vickerman, 1976

**Subclass:** Metakinetoplastina Vickerman, 2004

**Order:** Trypanosomatida (Kent, 1880) Hollande, 1952

**Family:** Trypanosomatidae (Doflein, 1901) Grobben, 1905

**Subfamily:** Phytomonadinae Yurchenko, Kostygov, Votýpka et Lukeš, 2015

**Genus:** *Phytomonas* Donovan, 1909

***Phytomonas lipae*** Frolov et Kostygov sp. n.

**Species diagnosis:** two morphotypes are present in host's salivary glands: 1) elongated promastigotes varying in size from 12 to 70 μm, and 2) small cells (6–11 μm) with no free flagella (endomastigotes). The anterior third of the body is widened, the posterior one is narrow and elongated; flagellum length is not greater than 1/3 of the promastigote's cell body; both nucleus and kinetoplast are located in the anterior part of the cell. The nucleus (2.5 μm ± 0.5 μm) is located in 2.9 μm ± 1.1 μm from the kinetoplast and 6.6 μm ± 1.6 μm from the anterior end. The compact kinetoplast (0.7 μm ± 0.1 μm × 0.2 μm ± 0.1 μm) is positioned in 1.7 μm ± 0.6 μm from the anterior end. The species can be identified by the sequences of 18S rRNA, gGAPDH, HSP83, and ITS1/ITS2 region (GenBank accession numbers: MK036047 –MK036051, MK050458 –MK050461, MK258191, and MK053634, respectively).

**Type host:** *Coreus marginatus* Linnaeus 1758 (Heteroptera: Coreidae). The xenotype collected on Russian dock *Rumex confertus* (Polygonaceae) is deposited at the Xenotypes’ Collection for Parasitic Protists in the Zoological Institute of the Russian Academy of Sciences (St. Petersburg, Russia).

**Location within host:** Present in the M1 (partly M2) midgut, hemolymph, and lumina of salivary glands, as well as within the cells of salivary glands.

**Type locality:** Novgorod Oblast, village Oksochi, 58°39' N, 32°47' E

**Type material:** The name-bearing type, a hapantotype, is a Giemsa-stained slide of the dissected salivary glands (isolate Cor4sg) it was deposited along with the axenic cultures Cor4, Cor49 and Cor203 in the Research Collection of Parasitic Protists of the Zoological Institute of the Russian Academy of Sciences (St. Petersburg, Russia).

**Etymology:** The specific name, *lipae*, honors Prof. Jerzy J. Lipa, who probably first observed promastigotes of this species in the dock bugs *Coreus marginatus*, but mistakenly identified them as a developmental stage of another trypanosomatid parasite, *Blastocrithidia raabei*, co-infecting the same host species [29].

## Discussion

Here we described a new species, *Phytomonas lipae*. It is apparently widespread in Eurasia: we documented its presence in two distant (over 2,200 km apart) populations of *Coreus marginatus* in northeastern Europe and southwestern Siberia. The insect host is a univoltine phytophagous bug, populating Eurasia and northern Africa. It may feed on many plants, but is mostly associated with seeds of docks and sorrels [54]. It is parsimonious to assume that these plants are hosts of *P*. *lipae*. Nevertheless, we could not detect the parasites by light microscopy in the seeds or sap of *Rumex confertus*, which were taken directly from nature.

### Phylogenetic position of the new species

The phylogenetic analysis implies rather early origin of the *Phytomonas lipae* lineage, right after that of *P*. *oxycarenus*. This demonstrates how understudied is the diversity of this genus. The inclusion of the two abovementioned species significantly changed the inferred picture of *Phytomonas* evolution. Recently, we brought to notice the paraphyly of phytomonads parasitizing latex, suggesting that such lifestyle was ancestral for the group [19]. However, in the light of the new data this hypothesis seems less plausible, as neither *P*. *lipae* nor *P*. *oxycarenus*, the two earliest branching members (Fig 2), live in lactiferous plants. As judged by the vectors' feeding habits [54], the former species should probably live in dock seeds. The bug host of the latter one feeds on various plants, but in Czechia (where we collected it), is strictly associated with lindens, predominantly *Tilia cordata*, and, apparently, feeds on their seeds and developing leaves [55,56]. Thus, both early diverging *Phytomonas* spp. probably live in plant seeds, suggesting a similar lifestyle for the ancestor of the genus. However, in order to better substantiate this, more data are needed on the life cycle of both species and overall diversity of the genus.

### Development in insects

Besides *P*. *lipae*, there are other three *Phytomonas* spp. with described developmental cycles in vectors: *P*. *serpens* (in *Phthia picta*, Coreidae), *P*. *nordicus* (in *Troilus luridus*, Pentatomidae), and *P*. *oxycareni* (in *Oxycarenus lavaterae*, Oxycarenidae) [19,21,28,57,58]. These parasites belong to different lineages within the genus and, therefore, knowledge of their phylogenetic relationships allows comparing their life cycles from the evolutionary perspective. All four species were documented in the intestine, hemolymph, and salivary glands of the infected insects. The ability to proliferate in the gut with formation of endomastigotes, which are dispatched with feces, was documented only for two of them–*P*. *serpens* and *P*. *nordicus* [28,57,58]. The intestinal stages of *P*. *oxycareni* have not been investigated in detail [21]. Promastigotes of *P*. *lipae* do not divide in the intestine and are restricted to the M1 and M2 segments of the midgut. However, this trait seems to be derived, given its state in *P*. *serpens*, *P*. *nordicus*, and trypanosomatids from other genera [59].

The mechanism of migration from midgut to hemolymph is unknown in any of *Phytomonas* spp. investigated thus far, but was scrutinized in *Trypanosoma rangeli* from *Rhodnius prolixus* (Reduviidae). This parasite penetrates vector's enterocytes with formation of parasitophorous vacuoles, which are then used as vehicles for the migration to the intestinal basal lamina, and after the disruption of the latter are released to the hemolymph [60]. Transient stages in hemolymph were described in all four *Phytomonas* spp. [19,21,28,57,58]. Here they reach the largest size (over 70 μm), and are characterized by a long flagellum, comparable in length to their twisted cell body.

The passage through the squamous coelomic epithelium surrounding salivary glands has not been studied in *P*. *oxycarenus*. In the other three species, it follows one of the two scenarios: the promastigotes of *P*. *serpens* and *P*. *lipae* migrate through intercellular space of the mesentery ([58] and this work), whereas *P*. *nordicus* invades cells of all tissues surrounding salivary glands (epithelial, muscle, and tracheal), and localizes in parasitophorous vacuoles [19]. The second variant appears derived, but in the absence of data on *P*. *oxycarenus* this cannot be properly justified.

The intracellular stages of the investigated *Phytomonas* spp. within salivary glands differ in the proliferative rates. Only singular (occasionally dividing in binary manner) promastigotes of *P*. *oxycareni* and *P*. *lipae* migrate through the cytoplasm in parasitophorous vacuoles ([21] and this work) and this is apparently the ancestral state. In two other species, *P*. *nordicus* and *P*. *serpens*, the migrating cells massively divide within parasitophorous vacuoles, forming pseudocysts with up to several dozen parasites [19,58]. In addition to this, the promastigotes of *P*. *serpens* can also migrate to the salivary gland lumen through intracellular space in the cubical epithelium [58].

In all four investigated species, micropopulations in the salivary gland lumen are morphologically heterogeneous and contain 2 main morphotypes: i) flagellated promastigotes of various sizes undergoing binary fission, and ii) small endomastigotes [19,57]. In the case of *P*. *oxycareni*, the second morphotype is not mentioned in the species description, but small (< 10 μm) aflagellated cells can be seen on SEM illustrations (Fig 3B in [21]). Similar cells in *P*. *lipae*, *P*. *serpens*, and *P*. *nordicus* have lengths of ~ 8 μm, ~ 6 μm, and ~ 12 μm, respectively ([19,57] and this work). Promastigotes of *P*. *nordicus* attach to the microvilli of the salivary gland epitheliocytes by a mechanism utilized by other trypanosomatids in the insect midgut or Malpighian tubules [32,37]. This appears to be a species-specific trait of this parasite, since in the three other investigated *Phytomonas* spp. these cells lie freely in the lumen ([21,58] and this work).

Summing it up, the new *Phytomonas* species, *P*. *lipae* is similar to the typical dixenous phytomonad species *P*. *serpens* and *P*. *oxycareni* in morphological traits and developmental program within its insect vector. It differs from the secondarily monoxenous *P*. *nordicus* in several essential respects, such as i) lack of the intestinal developmental stages, ii) careful passage through the coelomic epithelium, iii) lack of pseudocysts in the salivary gland epitheliocytes, and iv) inability of promastigotes to attach to microvilli. The three last traits are likely to be ancestral to all phytomonads.

## Supporting information

S1 Table GenBank accession numbers of all sequences used in phylogenetic analyses. (XLSX)

S2 Table Detailed morphometry of different cell types of *P*. *lipae*. (XLSX)
